# A rare case with melatonin‐induced on top of alcohol intoxication Brugada type 2 pattern

**DOI:** 10.1002/ccr3.8245

**Published:** 2023-11-16

**Authors:** Muad Abdi Hassan, Fatima Khalid Arbab, Obada Adel Alsakaji, Ahmad Dulli, Mohammad Abdow Abdow, Fatima Moulana Mohammed Jamal Ullah, Irfan Ullah Wali

**Affiliations:** ^1^ Medical Education Department Hamad Medical Corporation Doha Qatar; ^2^ Department of Medicine Hamad Medical Corporation Doha Qatar

**Keywords:** alcohol intoxication, Brugada pattern/phenocopy, Brugada syndrome, ECG changes, melatonin‐induced, sleep deprivation‐induced arrhythmias

## Abstract

Brugada syndrome (BrS), a genetically inherited ion channelopathy, has been linked to a considerable number of unexplained sudden cardiac deaths in patients without structural heart defects, and Brugada phenocopy (BrP) is a condition where there is an identical electrocardiogram (ECG) pattern to a congenital BrS, but this is due to other reversible etiologies. A 37‐year‐old male patient with a documented history of hypertension presented with vomiting after taking 43, 10 mg, melatonin pills and binge drinking locally made alcohol 2 days before. ECG showed right ventricular conduction delay with a “saddleback” appearance, with the J point elevated more than 2 mm and the terminal portion of the ST‐segment elevated more than 1 mm in leads V1 and/or V2. Which returned to normal after a few hours. The association between the use of melatonin and the finding of the Brugada pattern (BP) in a patient with normal heart structure or abnormal ECGs has been documented in much literature, and although no official melatonin dosage is recommended for adults, melatonin has been reported to cause and protect from arrhythmias through different mechanisms. In our patient, after alcohol intoxication was ruled out as a cause, melatonin was the only significant risk factor related to his ECG findings. The BP can be found in patients with otherwise normal heart structure and ECG records, and an overdose of melatonin, which is used as an over‐the‐counter sleep medication, was found to be a possible cause of finding this pattern in these patients after excluding other known causes.

## Graphical abstract

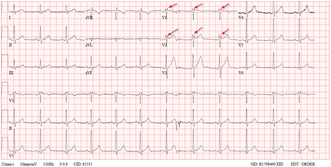

## 1 INTRODUCTION

Brugada syndrome (BrS), a genetically inherited ion channelopathy has been linked to a considerable number of unexplained sudden cardiac death (SCD) in patients without structural heart defects. Patients with the syndrome present with distinct patterns in electrocardiogram (ECG). Patients with BrS may experience syncope, seizures, and nocturnal agonal breathing caused by polymorphic ventricular tachycardia (PVT) or ventricular fibrillation (VF).^1^

A Brugada phenocopy (BrP) is a condition where there is an identical ECG pattern to a congenital BrS, but this is due to other reversible etiologies like electrolyte imbalance and some clinical conditions such as myocardial infarction.^2^ A quick resolution of the Brugada ECG pattern was noted upon treating the underlying factors.^3^

Here is a case that has never been reported in the literature review before which is Brugada pattern (BP) type 2 seen on an ECG following concomitant ethanol and melatonin overdose in a patient who had no known history of BrS before presented to the emergency department (ED).

## 2 CASE PRESENTATION

A 37‐year‐old male with a history of uncontrolled hypertension developed two episodes of vomiting an hour after taking 43 tablets of melatonin (10 mg) following an incident involving binge drinking locally brewed alcohol 2 days earlier. He stated that he smokes and experiences spells of sleep‐onset insomnia. The patient's vital signs were stable, the clinical exam, chest x‐ray, and lab results were unremarkable, and his blood pressure was within normal limits. Toxicology report for ethanol level was 35.7 which was an abnormal level. (Tables 1, 2, 3).

**TABLE 1:** Labs.

Lab tests	At admission	Normal values
WBC	8.3 × 10^3^/μL	4–10 × 10^3^/μL
RBC	5.3 × 10^6^/μL	4.5–5.5 × 10^6^/μL
Hb	15.5 g/dL	13–17 g/dL
Hct	43.9%	40–50%
MCV	82.8 fL	83–101 fL
MCH	29.2 pg	27–32 pg
MCHC	35.3 g/dL	31.5–34.5 g/dL
RDW‐CV	12.2%	11.6–14%
Platelet	294 × 10^3^/μL	150–410 × 10^3^/μL
PDW	12.8 fL	9.4–10.6 fL
Prothrombin time	11.8 s	9.4–12.5 s
INR	1.1	> 4.9 Critically high
APTT	30.1 s	25.1–36.5 s
Urea	3.2 mmol/L	2.5–7.8 mmol/L
Creatinine	84 μmol/L	62–106 μmol/L
Sodium	145 mmol/L	133–146 mmol/L
Potassium	4.1 mmol/L	3.5–5.3 mmol/L
Chloride	106 mmol/L	95–108 mmol/L
Bicarbonate	26 mmol/L	22–29 mmol/L
Calcium	2.29 mmol/L	8.6–10.3 mg/dL
Magnesium	0.78 mmol/L	0.70–1 mmol/L
Bilirubin	7 μmol/L	0–21 μmol/L
Total protein	72 g/L	60–80 g/L
Albumin	40 g/L	35–50 g/L
Alkaline phosphatase	66 U/L	40–29 U/L
ALT	25 U/L	0–41 U/L
AST	21 U/L	0–40 U/L
CK	72 U/L	39–308 U/L
CRP	<2.0 mg/L	0–5 mg/L

**TABLE 2:** Toxicology test.

Toxicology		
Ethanol	35.7mmol/L	> 44.9 critically high

**TABLE 3:** Vital signs.

Vital signs	At admission	Normal values
Temperature	36.9°C	35.5–38.5°C
Pulse rate	84 bpm	50–120 bpm
Respiratory rate	15 br/min	12–24 br/min
Systolic blood pressure	107 mmHg	100–180 mmHg
Diastolic blood pressure	78 mmHg	60–90 mmHg
Mean arterial pressure	88 mmHg	70 and 100 mmHg
SpO2	99%	

**FIGURE 1:**
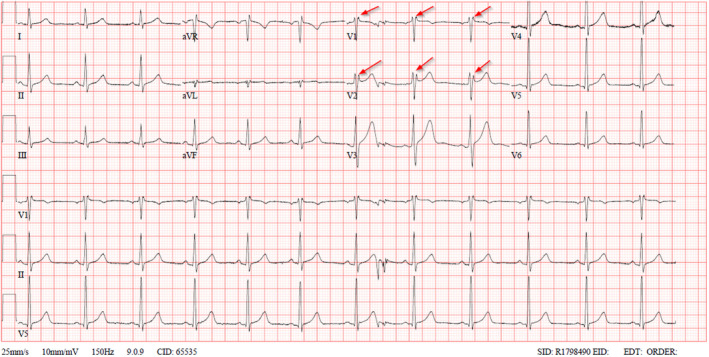
Brugada pattern type 2; saddleback appearance in the ST‐segment elevation more than 1 mm in leads V1 and/ or V2.

ECG showed signs of right ventricular conduction delay with a “saddleback” appearance, with the J point elevated more than 2 mm and the terminal portion of the ST‐segment elevated more than 1 mm in leads V1 and/ or V2. (Figure 1) As per these findings, the melatonin‐induced Brugada type 2 pattern was discovered.

**FIGURE 2:**
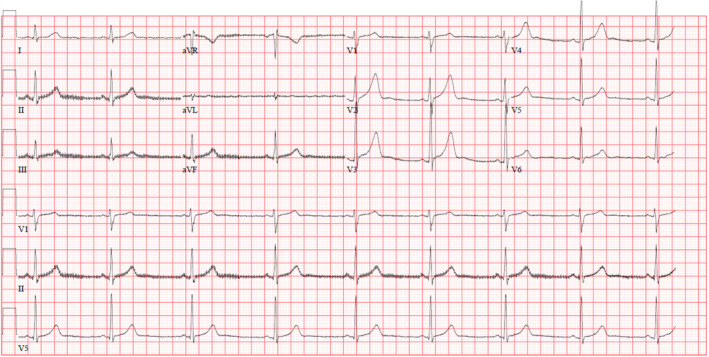
Normal electrocardiogram (ECG).

The major treatment strategies were folic acid, IV fluids, and thiamine. In addition, the patient said he had never considered harming himself or taking his own life, as he had grabbed the wrong bottle. His ejection fraction was 61%. The patient returned to his ECG baseline which was done 1 day after the previous one (Figure 2).

**FIGURE 3:**
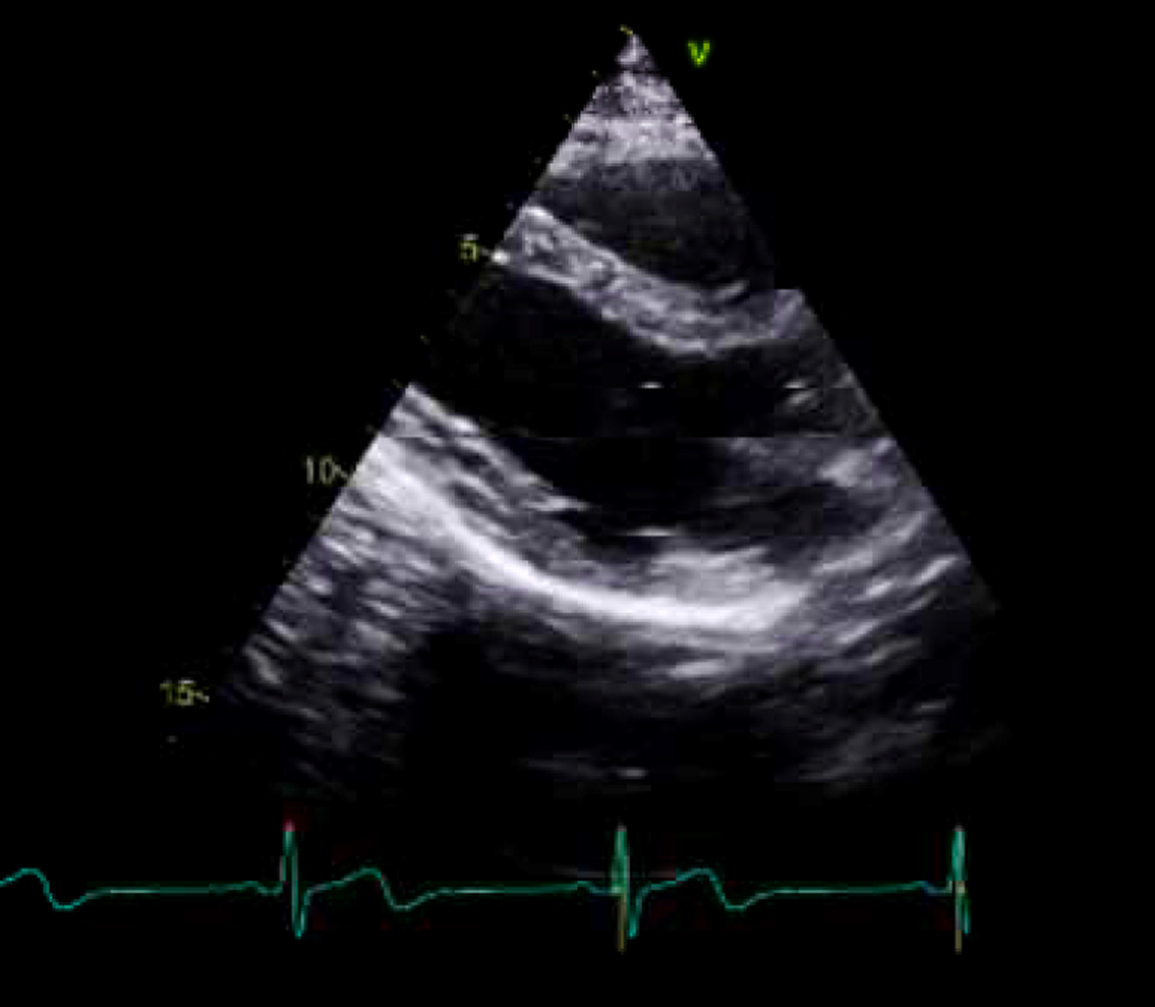
Normal echocardiogram (Echo).

Further investigation was done with an echocardiogram (Echo) which showed; LVEF at 61% with no regional wall motion abnormality and normal right ventricle function. (Figure **3**) The patient was declared free from psychiatry and cardiology acute points of view with a normal physical exam and was discharged the day after the admission. Upon discharge, the patient was booked to follow up with psychiatry and cardiology clinics.

## 3 DISCUSSION

This report is one of the few in the literature that discusses a possible link between the use of over‐the‐counter melatonin medication and the occurrence of a BP in a patient with a normal baseline ECG and no medical history apart from primary hypertension. It should be noted that in this case, the patient took an overdose of melatonin, exceeding the usual advised dosage, and also was binge drinking alcohol before the melatonin was presented to the ED.

BrS is the constellation of specific ECG patterns in addition to a history of sustained ventricular arrhythmias or the patient may experience syncope or SCD. Asymptomatic ECG patterns are termed BPs and these patterns can be persistently present or they are unmasked by several different stimuli. Some of the triggers for BP are infection, fever, toxins, and medications, especially flecainide, and procainamide.^4^

BrS is diagnosed with a combination of ECG patterns and clinical presentation. BP on ECG may present with three different patterns. Type 1 is a 2 mm coved ST segment or J point elevation followed by a negative T wave. Type 2 is the saddleback appearance of the T wave with at least 1 mm elevation of the ST segment or 2 mm elevation of the J point followed by a positive or biphasic T wave. Type 3 is either coved or saddleback appearance with >1 mm ST‐segment elevation. Three types of clinical criteria used to diagnose the disease include data from family history (ECG type 1 pattern at any age or SCD in family members with less than 45 years of age), ventricular arrhythmias (PVT or VF), and symptoms of arrhythmias (syncope, seizures).^5^

The patient initially presented with vomiting after binge drinking alcohol. Alcohol intoxication was ruled out, and instead, the focus was on the ingestion of melatonin pills which had been mentioned in their medical history. It was discovered that the alcoholic drink had been consumed before the melatonin pills and that the patient's symptoms had started right after ingestion of the pills, rather than after the alcoholic drink. This is important to highlight because alcohol drinking can precipitate BrS in patients who are known to have it, although the mechanism of precipitation is not yet fully understood. In addition, alcohol intake typically causes symptoms and ECG findings in patients with BrS. However, our patient experienced a complete reversal of ECG findings, which eventually returned to a normal baseline ECG, with a complete resolution of symptoms.

Melatonin on the other hand is a natural compound, specifically an indoleamine, produced by and found in different organisms including bacteria and eukaryotes.^6^

Interestingly, sleep medications containing melatonin can potentially induce ventricular arrhythmias in structurally normal hearts. Reports in the literature mainly argue for a protective effect of melatonin against arrhythmias. This putative antiarrhythmic effect has been implicated to occur through indirect mechanisms, as a sleep medication, it might be able to alleviate “sleep deprivation–induced arrhythmias.” In addition, by reducing the sympathetic tone, melatonin can also reduce the arrhythmia burden caused by sympathetic predominance.^7^

There is no official recommended melatonin dosage for adults, but a range of 1–5 mg is considered within normal ranges. The possible side effects associated with melatonin overdose include headache, dizziness, nausea, and daytime drowsiness. Other, less common side effects might include: Vivid dreams or nightmares, short‐term feelings of depression, irritability, stomach cramps, diarrhea, constipation, decreased appetite, urinary incontinence at night, increased risk of falls, increased risk of seizures, confusion or disorientation, reduced alertness, but there are no enough data on the possible effects on the heart's rhythm.

## 4 CONCLUSION

This case presents the association between BP, which represents specific ECG findings in a patient with a structurally normal heart and no symptoms, in a patient that came with a history of consuming 43 pills of melatonin 2 days after binge drinking alcohol, the thing that was ruled out as a cause after the level of ethanol came out normal, and after few hours, in which the patient was treated conservatively, the ECG returned to normal and the other investigations that were done to the patient were normal as well. Although there is no well‐known association between melatonin and BP specifically, it was shown in multiple reports that melatonin can be associated with ventricular arrhythmias in structurally normal hearts through “sleep deprivation–induced arrhythmias.” In addition, by reducing the sympathetic tone, melatonin can also reduce the arrhythmia burden caused by the sympathetic predominance.

## AUTHOR CONTRIBUTIONS

**Muad Abdi Hassan:** Data curation; project administration; writing – original draft; writing – review and editing. **Fatima Khalid Arbab:** Writing – original draft. **Obada Adel Alsakaji:** Writing – original draft. **Ahmad Dulli:** Writing – original draft. **Mohammad Abdow Abdow:** Writing – original draft. **Fatima Moulana Mohammed Jamal Ullah:** Writing – original draft. **Irfan Ullah Wali:** Supervision; writing – review and editing.

## FUNDING INFORMATION

This case report was not funded.

## CONFLICT OF INTEREST STATEMENT

The authors have declared that no competing interests exist.

## ETHICS STATEMENT

The article describes a case report. Therefore, no additional permission from our ethics committee was required.

## CONSENT

Written informed consent was obtained from the patient to publish this report in accordance with the journal's patient consent policy.

## Data Availability

All data generated or analyzed during this study are included in this published article.
